# Early range of motion results in good elbow function following conservative treatment of non-displaced radial head fractures

**DOI:** 10.1007/s00402-024-05293-7

**Published:** 2024-04-13

**Authors:** Philipp Egenolf, Nadine Ott, Tamara Babasiz, Michael Hackl, Lars-Peter Mueller, Sebastian Wegmann

**Affiliations:** Department of Orthopaedic and Trauma Surgery, Faculty of Medicine, University of Cologne, University Hospital Cologne, Cologne, Germany

**Keywords:** Radial head, Fracture, Conservative treatment, Elbow function, Return to work

## Abstract

**Introduction:**

The aim of this study was to evaluate the range of motion (ROM), elbow function and predictors for good elbow function after conservative treatment of non-displaced radial head fractures.

**Material and methods:**

All patients with non-displaced radial head fractures (displacement < 2 mm), that were diagnosed between January 1st 2017 and December 31st 2021 in a level I trauma center, were included in this retrospective case series and the charts were evaluated for ROM and elbow function. Elbow function was categorized as “good” or “bad” depending on the ROM measured defined by Morrey et al. Overall, 73 patients (33 male, 40 female) with an average age of 38 years (+/- 13 years) could be included.

**Results:**

Conservative treatment had good clinical results for ROM and elbow function. After 6 weeks mean flexion was 131° (SD 13°), extension 8° (SD 7°), Pronation 83° (SD 11°) and Supination 83° (SD 13). Patients with a good elbow function after one week showed a good elbow function after completing the treatment.

**Conclusions:**

A clinical assessment after one week should always be performed and the study showed that it is a good predictor for good elbow function. In cases of bad elbow function further controls should be considered.

**Supplementary Information:**

The online version contains supplementary material available at 10.1007/s00402-024-05293-7.

## Introduction

Fractures of the radial head are common, accounting for 1/3 of all fractures of the elbow and are usually caused by a fall on the outstretched arm with the elbow in pronation and partial flexion or rarely due to direct trauma [1, 2]. Approximately 80% of all radial head fractures occur in adults with a male to female ratio of 1:2 [3]. The most commonly used classification is the Mason classification [4]. He assigned radial head fractures to three different categories based on displacement and comminution. (Table 1) A fourth category was added by Johnston for radial head fractures associated with elbow dislocation [5, 6]. Based on conventional radiographs in three planes (a.p., lateral and Coyle´s view) the fractures are assigned to the above-mentioned categories and a therapy recommendation is formulated. Depending on the fracture type, dislocation and additional injuries the therapeutic range for Mason type II-IV fractures ranges from non-operative treatment to open reduction and internal fixation up to radial head resection and implantation of a radial head prothesis. Non-displaced or minimally displaced (< 2 mm) Mason type I fractures are with 64% of all radial head fractures the most common ones and are generally treated conservatively [7, 8]. A short period of immobilization (approx. 1 week) in a cast is followed by early functional treatment with free range of motion. Load bearing should be avoided for 6 weeks, while active and active-assisted elbow movement and forearm rotation is allowed [9]. In the current literature only few studies attend to Mason type I fractures, but they show that in > 90% a good to very good results can be expected [8, 10–12]. In a multicenter study Hackl et al. could demonstrate, that complications following radial head fractures are correlated to their severity and hence their classification and that for Mason type I and II fractures stiffness and symptomatic osteoarthritis were the most common reasons for a revision [13]. In his study Morrey described, that an elbow flexion of 100 degrees (30–130°) and a forearm rotation of 100 degrees (50° pro-and supination) were necessary for most activities of daily living [14]. Burkhart et al. recommended early functional therapy following Mason type I fractures and hypothesized, that the clinical examination und range of motion after cast removal would be a good indicator for the further course of treatment and to detect treatment failure [15]. In 90% of the cases a good clinical outcome is expected, and literature recommends clinical controls after 1,3 and 6 weeks. With this study we aimed to evaluate, if regular controls are necessary and to elaborate indicators for good elbow function.

**Table 1:** Average range of motion (ROM) values and standard deviation (± SD)

	Initial presentation (ROM_0_)		1 week follow up (ROM_1_)		6 week follow up (ROM_6_)	
Values	Average (± SD)	Sig. (MEF_6_)	Average (± SD)	Sig. (MEF_6_)	Average (± SD)	
Flexion	110° (± 23°)	0.181	117° (± 16°)	0.011	131° (± 13°)	
Extension *	24° (± 19°)	0.116	17° (± 12°)	0.057	8° (± 7°)	
Pronation	72° (± 22°)	0.165	73° (± 20)	0.127	83° (± 11°)	
Supination	72° (± 21°)	0.127	72° (± 21°)	0.091	83° (± 13°)	

* (deficit to neutral). Significance (Sig.) is given for correlation of the type of freedom to its MEF_6_

## Methods

### Population and therapy algorithm

All patients with a radial head fracture Mason type I, which were diagnosed between January 1st 2017 and December 31st 2021 in our level I trauma center, were included in this retrospective case series. Inclusion criteria were isolated radial head fractures Mason type I, which were radiographically diagnosed. Radiography consisted of conventional x-ray of the elbow in two planes as well as a radial head view. Exclusion criteria where additional fractures of the elbow joint, age younger than 18 years and loss to follow up at the defined routine intervals of 1 and 6 weeks after injury.

Resulting from our standard operating procedure, all patients initially received an upper arm cast in 90° flexion for up to one week. CT-imaging was performed within the first seven days, predominantly within the first two days. In cases of fracture displacement larger than 2 millimetres (mm), a CT- scan was performed to further evaluate the fracture, and if necessary, patients were admitted to surgery and consequently excluded from this observational study. The first follow-up was performed one week after diagnosis. It included a radiological as well as clinical assessment. If articular fracture displacement showed increasing displacement, patients were also admitted to surgery and excluded. If no displacement occurred until the follow-up after one week, patients’ casts were switched to a two-dimensional elbow brace with a mobile joint. The brace allowed for full range of motion (ROM) of the elbow while protecting the joint from varus/valgus force. Patients were advised to perform repetitive physiotherapy following the overhead motion-protocol [17] and to avoid forced extension and flexion for the upcoming 5 weeks.

**Fig. 1:**
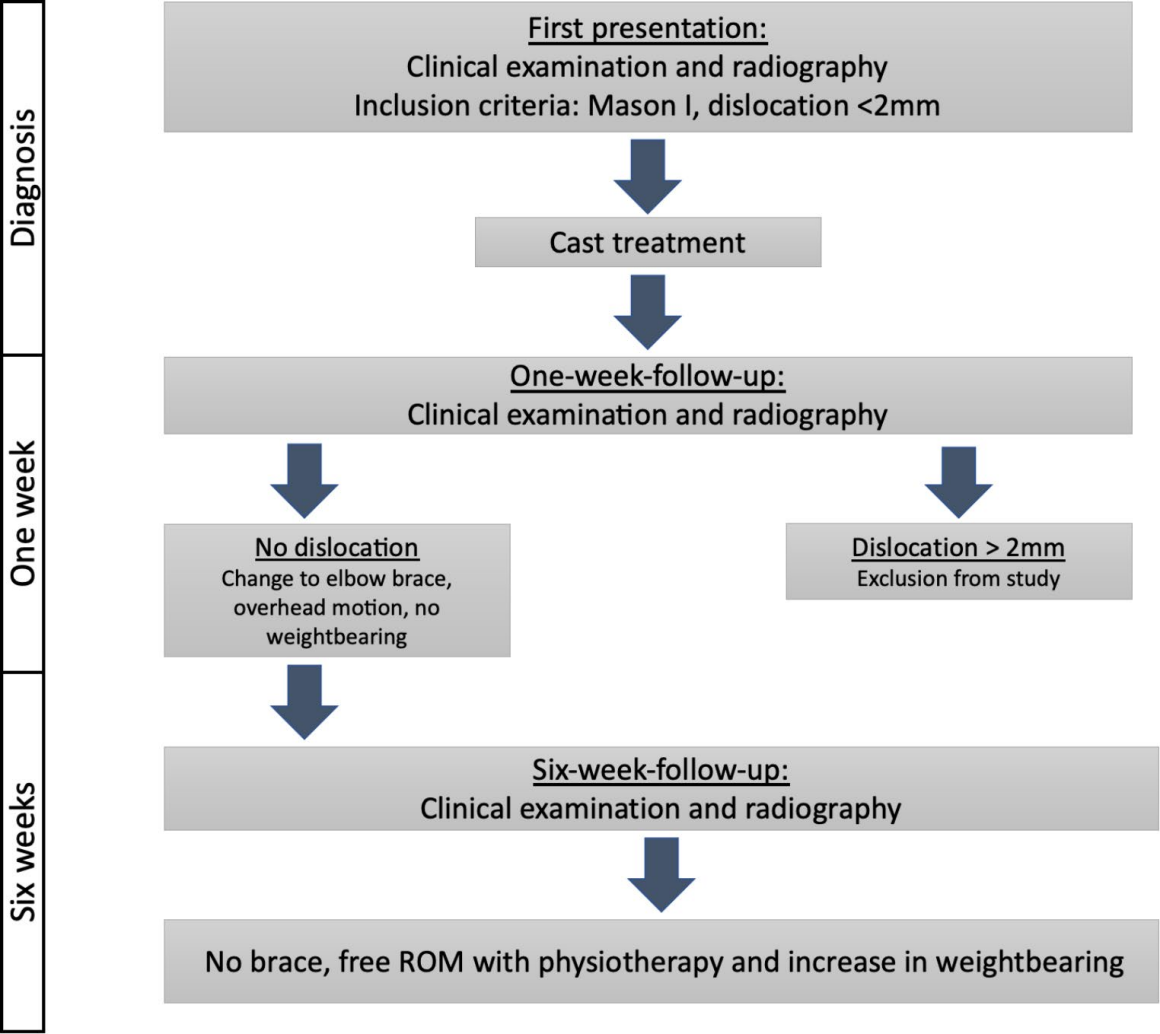
Therapy algorithm of non-displaced radial head fractures including inclusion and exclusion criteria

Final clinical and radiological assessment was performed 6 weeks after initial diagnosis. Regularly, the brace was removed hereafter and patients were encouraged to increase weight bearing and to fully use the injured arm. (Fig. 1)

All the clinical assessments included a detailed examination of the elbow but also the forearm to exclude injuries to the distal radioulnar articulation (DRUA). In the observed population, no patients suffered from an injury to the DRUA.

### Clinical assessment and evaluation

Clinical assessment included range of motion for flexion and extension of the elbow and pronation and supination of the forearm. For statistical analysis, lack of extension towards neutral was noted instead of the patient’s ability to extend further than neutral.

As an additional descriptive tool, patients’ range of motion was evaluated following the suggestion of Morrey elbow function (MEF) [14]. Following their recommendation, range of motion was graded into *good elbow function* (GEF) and *bad elbow function* (BEF). GEF was defined as 130° (degrees) for flexion (GEF_F_) and less than 30° extension deficit (GEF_E_). Pronation and supination had to be at least 50° each for pronation (GEF_P_) and supination (GEF_S_).

### Outcome parameters

Primary descriptive outcome measures were absolute ROM as well as MEF.

To evaluate if ROM is predictive for MEF after 6 weeks (MEF_6_), each ROM_0_- and ROM_1_-parameter was evaluated depending on its MEF_6_ (GEF, BEF).

### Statistical analysis

The normality assumption was checked visually and by using the Kolmogorov-Smirnov test. Hereby no normal distribution could be assumed.

Development of elbow ROM during the observed 6-week time was evaluated via Wilcoxon rank test.To evaluate ROM’s predictive value, each parameter’s MEF_6_ was correlated to its ROM_0_ and ROM_1_. Assuming a predictive value of the early range of motion, one-sided Spearman correlation was applied. Level of significance was set at *p* = 0.05. Statistical analysis was performed using SPSS 28.0 (IBM SPSS Statistics, Chicago, IL, USA).

## Results

Overall, a total of 141 patients (69 male, 72 female) were initially diagnosed with a radial head fracture, classified as Mason I. 66 patients were not cohesively followed at the defined time-periods and were therefore excluded from this study. Two patients were initially diagnosed with a sole radial head fracture but following computed tomography (CT) and clinical evaluation lead to surgery instead. One patient had an interposed fragment in the proximal ulnoradial joint with a rotation block. Supination was limited to a maximum of 45°. The fracture was arthroscopically fixed. The other patient’s CT, two days after trauma, revealed an articular fracture displacement of > 2 mm. He was also admitted to surgery.

No patients were excluded due to fracture dislocation larger than 2 mm at the follow-ups.

After all, 73 patients (33 male, 40 female; right arm: 35, left arm: 38) were included in this study. Average age was 38 years (+/- 13 years).

### Clinical outcome measures

Average values for ROM including standard deviation and total values are given in Table 1. Distribution of MEF values divided into GEF and BED for extension, flexion, pronation and supination are reported in Table 2. While flexion after 6 weeks was not valued *good* (GEF) in 22 patients, only one patient did not reach GEF regarding the other degrees of freedom.

**Table 2:** Distribution of MEF values, separated into *good elbow function* (GEF) and *bad elbow function* (BEF)

MEF	GEF	BEF	GEF	BEF	GEF	BEF
Flexion	18 (24,66%)	55 (75,34%)	22 (30,14%)	51 (68,49%)	51 (69,86%)	22 (30,14%)
Extension	45 (61,64%)	28 (38,36%)	67 (91,78%)	6 (8,22%)	72 (98,63%)	1 (1,37%)
Pronation	61 (83,56%)	12 (16,44%)	64 (87,67%)	9 (12,33%)	72 (98,63%)	1 (1,37%)
Supination	62 (84,93%)	11 (15,07%)	61 (83,56%)	12 (16,44%)	72 (98,63%)	1 (1,37%)

Spearman test revealed no correlation between ROM_0_ with MEF_6_ of either flexion, extension, pronation or supination (*p* > 0.05; Table 1). Comparing ROM_1_ to MEF_6_ however, ROM_1 − F_ however correlated significantly with MEF_6 − f_ (*p* = 0.011; Table 1).

Comparing ROM_0_ to ROM_1_ showed a significant improvement solely of flexion (*p* = 0.01), while extension, pronation and supination values did not increase significantly (Table 3). However, all degrees of joint freedom (e.g. flexion) improved significantly from ROM_0_ to ROM_6_ and ROM_1_ to ROM_6_ (Table 3).

**Table 3:** Development of ROM values between the initial examination (ROM_0_) and follow up examinations (ROM_1_; ROM_6_). Significance (p) is given as result of Wilcoxon Test

Values	Significance (p)		
Interval	ROM_0_ – ROM_1_	ROM_0_ – ROM_6_	ROM_1_ – ROM_6_
Flexion	0.010	0.001	0.001
Extension	0.024	0.001	0.001
Pronation	0.981	0.001	0.001
Supination	0.790	0.001	0.001

## Discussion

Our study has shown that our treatment algorithm for Mason type I fractures overall had a good clinical outcome with very good elbow function after 6 weeks of conservative therapy with early mobilization and using the overhead motion protocol. This result is in conformity with other studies conducted and in line with the current recommendations for the treatment of radial head fractures Mason type I [12, 18, 19]. 

Furthermore, the study has shown that ROM evaluated one week after the injury gave more significant results predicting the overall outcome compared to initial assessment. In the injury moment a precise evaluation is often difficult due to swelling and pain. The immobilization in the cast is intended to reduce these factors. This may be the reason why results after one week showed better potential to properly display limitations of movement. Therefore, we recommend a time-displaced reevaluation at one week post trauma. Additionally, further controls, like suggested in the literature, did not prove to be beneficial and should therefore be avoided.

In our study reduced flexion was the main problem after six weeks, other planes of motion had a satisfactory outcome. However, a full range of motion is not essential for performance of all activities of daily living. A functional arc of flexion and extension is said to range from 30–130° and 50° of pro- and supination each to perform 90% of daily activities [20]. In our study only one patient had a bad elbow function for supination after one week, which was the indicator for CT-imaging. Revealing an interposed fragment, this patient underwent open reduction and internal fixation. Therefore, BEF for pronation and supination as well as extreme outliers in flexion/extension should give reason to worry at a one week assessment. In these cases, computed tomography should be consulted and operative therapy could be chosen [21]. 

This study has several limitations. First, we experienced a high loss-to-follow-up rate. While this was mainly due to the retrospective design of the study, a selection bias can not be ruled out. The second limitation is the relatively short follow up, which limits the evaluation of potential worsening of initially good elbow function. Lastly, only range of motion was evaluated as outcome parameter due to the retrospective nature of this study. Although MEF evaluates the range of motion from a clinical point of view, further elbow function scores e.g. Mayo Elbow Performance Index could give additional insights. This should be considered in future prospective studies.

## Conclusion

Cast immobilization followed by free range of motion with orthotic assistance shows satisfactory short-term outcomes of non-displaced radial head fractures. Clinical evaluation after one week is a good predictor for potential complications of conservative therapy and more predictive than the initial evaluation. If a one week evaluation shows good clinical results and a secondary dislocation of the fracture can be excluded, further evaluations within the 6 weeks of therapy don’t seem to be necessary. Flexion is most likely not to reach sufficient range of motion after 6 weeks.

### Electronic supplementary material

Below is the link to the electronic supplementary material.

Supplementary Material 1
