# Supplementary material for: Early range of motion results in good elbow function following conservative treatment of non-displaced radial head fractures
Source: Arch Orthop Trauma Surg. 2024 Apr 13;144(5):2165–9. doi: 10.1007/s00402-024-05293-7 (PMC11093790; doi:10.1007/s00402-024-05293-7)
Supplement: Supplementary file 1 — Supplementary Material 1 [file 402_2024_5293_MOESM1_ESM.docx]

Early range of motion results in good elbow function following conservative treatment of non-displaced radial head fractures

Philipp Egenolf^1^, Nadine Ott^1^, Tamara Babasiz^1^, Michael Hackl^1^, Lars-Peter Mueller^1^, Sebastian Wegmann^1^

^1^ University of Cologne, Faculty of Medicine and University Hospital Cologne, Department of Orthopaedic and Trauma Surgery, Germany

Correspondence

Dr. med. Philipp Egenolf

University Hospital of Cologne, Department for Orthopaedic and Trauma Surgery

Joseph-Stelzmann-Str. 24, 50931 Cologne, Germany

ORCID ID: https://orcid.org/0000-0002-4451-2986

E-Mail: philipp.egenolf@uk-koeln.de

Declarations:

Author contributions:

All authors contributed to the study conception and design. Material preparation, data collection and analysis were performed by Philipp Egenolf, Tamara Babasiz and Sebastian Wegmann. The first draft of the manuscript was written by Philipp Egenolf and Sebastian Wegmann and all authors commented on previous versions of the manuscript. All authors read and approved the final manuscript.

Compliance with Ethical Standards:

The authors have no competing interests to declare that are relevant to the content of this article.

Ethical approval:

This study was submitted to the local ethics committee, which did not see any obligation to advise due to the professional law requirements. Written consent was waived due to the retrospective nature of this investigation. All radiological diagnostics were clinically indicated and not performed for study purposes.

10.06.2023
